# Synthesis and Antimicrobial Activity of 6-Thioxo-6,7-dihydro-2*H*-[1,2,4]triazino[2,3-*c*]-quinazolin-2-one Derivatives

**DOI:** 10.3797/scipharm.1402-10

**Published:** 2014-03-24

**Authors:** Inna S. Nosulenko, Olexii Yu. Voskoboynik, Galina G. Berest, Sergiy L. Safronyuk, Sergiy I. Kovalenko, Oleksandr M. Kamyshnyi, Nataliya M. Polishchuk, Raisa S. Sinyak, Andrey V. Katsev

**Affiliations:** ^1^ Department of Organic and Bioorganic Chemistry, Zaporizhzhia State Medical University, Mayakovsky ave., 26, 69035, Zaporizhzhia, Ukraine.; ^2^ Department of Pharmacognosy, Pharmaceutical chemistry and Technologies, Department of post-graduate education, Zaporizhzhia State Medical University, Mayakovsky ave., 26, 69035, Zaporizhzhia, Ukraine.; ^3^ Department of Pharmacy, Crimean State Medical University, 95006, Simferopol, Ukraine.; ^4^ Department of Microbiology, Virology and Immunology, Zaporizhzhia State Medical University, Mayakovsky ave., 26, 69035, Zaporizhzhia, Ukraine.; ^5^ Department of General Chemistry, Crimean State Medical University, 95006, Simferopol, Ukraine.

**Keywords:** Synthesis, Potassium salt, 6-Thioxo-6,7-dihydro-2*H*-[1,2,4]triazino[2,3-*c*]quinazolin-2-one derivatives, Antimicrobial activity

## Abstract

Potassium 8-R^1^-9-R^2^-10-R^3^-3-R-2-oxo-2*H*-[1,2,4]triazino[2,3-*c*]quinazoline-6-thiolates **2.1–2.26** were synthesized via cyclocondensation of 6-R-3-(3-R^1^-4-R^2^-5-R^3^-aminophenyl)-1,2,4-triazin-5-ones **1.1–1.26** with carbon disulfide, potassium hydroxide, and ethanol or with potassium *O*-ethyl dithiocarbonate in 2-propanol. The corresponding thiones **3.1–3.26** were obtained by treatment of **2.1–2.26** with hydrochloric acid. It was found that the nature of the substituents in positions 3, 4, and 5 of the corresponding 6-R-3-(3-R^1^-4-R^2^-5-R^3^-aminophenyl)-1,2,4-triazin-5-ones were affected on the terms of the reaction. The structures of compounds were proven by a complex of physicochemical methods (^1^H, ^13^C NMR, LC–MS, and EI-MS). The results of the antibacterial and antifungal activity assay allowed the determination of the high sensitivity of *Staphylococcus aureus* ATCC 25923 (MIC 6.25–100 μg/mL, MBC 12.5–200 μg/mL) to the synthesized compounds.

## Introduction

Native and synthetic quinazoline derivatives are some of the priority objects of investigation in current organic and pharmaceutical chemistry. A lot of attention over the mentioned class of compounds caused by the broad potential of its chemical modification is aimed at the synthesis of the novel perspective potential medications [1–4]. Recent publications describe the synthesis and biological activity of the [1, 2, 4]triazino[2, 3-*c*]-quinazoline series [5–13]. It is known that the introduction of a thio-group in position 6 of the [1, 2, 4]triazino[2, 3-*c*]quinazoline system allows the obtainment of compounds with significant cytotoxic action against *Photobacterium leiognathi* [9, 11–13] and antimicrobial action against *Staphyloccocus aureus* and *Aspergillus niger* [8–13]. The following structure modification of 6-thio-3-R-2*H*-[1, 2, 4]triazino[2, 3-*c*]quinazoline-2-ones by synthesis of S-substituted derivatives enabled the obtainment of compounds with anticancer action [9, –13]. Authors noted that the above-mentioned type of action depends substantially on the nature of the pharmacophore fragment in positions 3 and 6. Adhering to the previously developed strategies of the target synthesis of chemotherapeutic agents, we decided to realize further structure modifications of 6-thio-3-R-2*H*-[1, 2, 4]triazino[2, 3-*c*]-quinazoline-2-ones by the introduction of halogen and methyl substituents in positions 8, 9, 10 and study the antibacterial and antifungal activities of the synthesized compounds. Thus, this work aimed to study the influence of substituents (alkyl and halogen groups) in 6-R-3-(2-aminophenyl)-2*H*-[1, 2, 4]triazin-5-ones on the cyclocondensation process and on the antibacterial and antifungal activities of the synthesized compounds.

## Results and Discussion

### Chemistry

As starting compounds we used 6-R-3-(3-R^1^-4-R^2^-5-R^3^-2-aminophenyl)-1,2,4-triazin-5-ones (**1.1–1.26**), which were obtained according to known protocols, namely by nucleophilic cleavage of the pyrimidine fragment in 3-R-8-R^1^-9-R^2^-10-R^3^-2*H*-[1, 2, 4]triazino[2, 3-*c*]quinazolin-2-ones or hydrazinolysis of 2-aryl-[(3*H*-quinazolin-4-ylidene)hydrazono]acetic acids esters [14]. Synthesis of potassium thiolates **2.1–2.26** was performed by the interaction of initial compounds **1.1–1.26** with sulfur disulfide, ethanol, and potassium hydroxide in ethanol (Method A) or potassium *O*-ethyl dithiocarbonate in 2-propanol [8] **1.1–1.26** (Method **B**, Scheme 1). The products of the mentioned cyclocondensation were the individual compounds **2.1–2.26** with yields of 64-99%. Method B had some advantages: ease of execution, health safety, high yields, and purity of the final products. To prove the structure, the synthesized thiolates **2.1–2.26** were transformed in the corresponding thions **3.1–3.26** by acidifying the water solutions of the mentioned potassium salts with hydrochloric acid to pH 2–3. We noted that the substituents in positions 3, 4, and 5 in the corresponding 6-R-3-(2-aminophenyl)-1,2,4-triazino-5-ones (**1.1–1.26**) significantly affected the reaction process. So, according to LC-MS (APCI), the initial compounds, which contained the substituents chlorine, bromine, or iodine that were needed, increased the duration of heating to 8–10 hours.

**Sch. 1.**
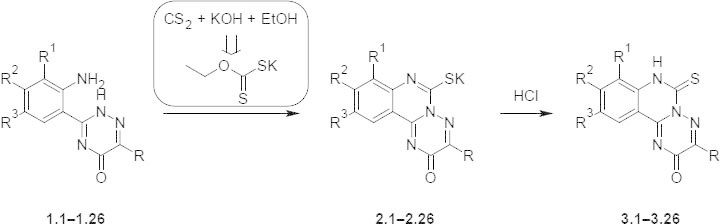
Synthesis of the potassium 3-R-8-R^1^-9-R^2^-10-R^3^-2-oxo-2*H*-[1, 2, 4]triazino[2, 3-*c*]-quinazoline-6-thiolates **2.1–2.26** and 3-R-8-R^1^-9-R^2^-10-R^3^-6-thioxo-6,7-dihydro-2*H*-[1, 2, 4]triazino[2, 3-*c*]quinazolin-2-ones **3.1–3.26**

The purity of the synthesized compounds was confirmed by LC-MS (APCI) data, and the structure by IR-, ^1^H-, ^13^C NMR-, mass-spectra, and elemental analysis. In the LC-MS (APCI) spectra of **3.1–3.26**, the positive ions [M+1] and [M+3] (sulfur isotope) were observed. The value of the molecular mass coincided with the expected for the synthesized compounds. The mass-spectra (EI) of thiones **3.1–3.4**, **3.8** had some traits and substantial differences from the other heteroaromatic sulfides. In this case, molecular ion fragmentation under the electron impact pass on the C(2)–C(3) and N(4)–N(5) bond followed by the elimination of the nitrile radical and formation of an ion with m/z 203 had the highest intensity (96.7–100%). The mentioned ion underwent cleavage with the elimination of S, SH, CNS, CHNS, and CNO fragments and formation of an ion with m/z 171, 170, 145, 144, 161 with the corresponding intensity.

Our attempt to record the NMR spectral data for potassium 8-R^1^-9-R^2^-10-R^3^-3-R-2-oxo-2H-[1, 2, 4]triazino[2, 3-*c*]quinazoline-6-thiolates in DMSO-*d*_6_, CDCl_3_, and D_2_O failed. We considered this as a consequence of the exchange processes, tautomeric transformations, and insufficient solubility. However, for the evaluation of the mentioned compound structure, we converted them into thiones **3.1–3.26** and recorded all of the necessary spectral data to prove their structures. So, as we considered, the confirmation of the structures **3.1–3.26** in combination with the IR and elemental analysis data, proved the structures of compounds **2.1–2.26.**

The ^1^H-NMR-spectra of compounds **3.1–3.26** are characterized by singlet signals of the thioamide group proton at 14.22–13.79 ppm and aromatic protons of the triazinoquinazoline system with corresponding chemical shifts [16]. The appearance at 171.05–168.79 ppm and 158.68-160.2 ppm of the characteristic signals of deshielded C-2 and C-6 carbons in the ^13^C NMR-spectra of compounds **3.1**, **3.3**, **3.4**, and **3.8** confirm the formation of the new heterocyclic system. Also in the ^13^C NMR-spectra of the mentioned compounds, the signals of the corresponding aliphatic carbons are present. As we considered, the used physicochemical methods completely confirm the structure of the synthesized compounds.

The IR-spectra of compounds **3.1–3.26** are characterized by intensive absorption at 1767–1590 cm^-1^, which correspond to vibrations of the C=S and C=O groups and substantially differentiate them from their initial compounds. At the same time in the IR-spectra of thiolates (**2.1–2.26**), the mentioned signals were subjected to the hypsochromic shift which may be explained by the formation of an ion bond. The vibrations of C=C bonds of the aromatic fragment at 1589–1468 cm^-1^ of the non-plate deforming vibrations of the =C-H bond at 850–666 cm^-1^ and intensive signals at 2960–2850 cm^-1^, caused by the symmetric and asymmetric vibrations of CH_2_ and CH_3_ groups, are also present in the IR-spectra of the compounds **2.1–2.26** and **3.1–3.26**. The IR-spectra of compounds, which contain halogen intensive signals of vC-F, vC-CI, vC-Br, vC-l, are also present.

**Tab. 1.** Antimicrobial activities of potassium 8-R^1^-9-R^2^-10-R^3^-3-R-2-oxo-2H-[1, 2, 4]triazino[2, 3-*c*]quinazoline-6-thiolates

Comp.	R	R^1^ = R^2^ = R^3^ = H (if not specified)	Investigated strains							
			*E. coli*		*S. aureus*		*P. aeruginosa*		*C. albicans*	
			MIC μg/mL	MBC μg/mL	MIC μg/mL	MBC μg/mL	MIC μg/mL	MBC μg/mL	MIC μg/mL	MBC μg/mL
2.1	CH_3_		100	100	50	200	50	200	100	100
2.2	C_6_H_5_		100	200	25	100	100	200	50	100
2.3	C_6_H_4_(CH_3_)-*p*		50	100	12.5	100	100	200	100	100
2.4	C_6_H_4_(CH_3_)_2_-*3,4*		100	100	100	200	50	100	50	100
2.5	C_6_H_4_(C_2_H_5_)-*p*		100	100	12.5	50	100	100	50	100
2.6	C_6_H_4_(*i*-C_3_H_7_)-*p*		100	100	25	50	50	100	50	100
2.7	C_6_H_4_(*tert*-C_4_H_9_)-*p*		50	100	6.25	12.5	50	100	100	100
2.8	C_6_H_4_(OCH_3_)-*p*		100	200	100	200	50	200	50	100
2.9	C_6_H_4_(OC_2_H_5_)-*p*		100	100	25	100	50	100	50	100
2.10	C_6_H_4_F-*p*		50	50	12.5	50	50	100	50	100
2.11	C_6_H_5_	R^1^ = CH_3_	100	100	25	100	50	200	50	100
2.12	C_6_H_5_	R^2^= F	100	200	6.25	25	100	200	50	50
2.13	C_6_H_4_F-*p*	R^2^= F	100	200	6.25	25	50	100	50	100
2.15	C_6_H_5_	R^3^ = Cl	100	200	6.25	100	50	100	50	50
2.16	C_6_H_4_(CH_3_)-*p*	R^3^ = Cl	100	200	6.25	100	100	100	50	100
2.17	C_6_H_4_(OCH_3_)-*p*	R^3^ = Cl	100	200	12.5	100	50	100	100	100
2.18	C_6_H_4_F-*p*	R^3^ = Cl	100	200	12.5	50	100	200	50	50
2.19	C_6_H_4_F-*p*	R^1^ = Br	100	200	12.5	25	50	100	50	100
2.20	C_6_H_4_F-*p*	R^2^ = Br	100	200	6.25	25	100	200	50	100
2.21	C_6_H_5_	R^3^ = Br	100	200	6.25	50	50	200	50	50
2.22	C_6_H_4_(CH_3_)-*p*	R^3^ = Br	100	200	12.5	50	50	100	50	50
2.23	C_6_H_4_F-*p*	R^3^ = Br	100	200	6.25	25	50	200	50	50
2.24	C_6_H_4_(OCH_3_)-*p*	R^3^ = Br	100	200	12.5	200	100	100	100	100
2.25	C_6_H_4_F-*p*	R^3^ = 1	100	200	6.25	200	100	200	50	50
2.26	C_6_H_4_(OCH_3_)-*p*	R^3^ = l	100	200	50	200	100	100	50	50
Trimethoprim		–	50	50	31.2	62.5	62.5	125	62.5	125
Nitrofural		–	1.5	–	6.25	–	6.25	–	25.0	–

### Antimicrobial Activities

Results of the conducted microbiological screening showed that 3-R-8-R^1^-9-R^2^-10-R^3^-6-thioxo-6,7-dihydro-2*H*-[1, 2, 4]triazino[2, 3-*c*]quinazolin-2-ones (**3.1–3.26**) exhibit moderate inhibitory activity (MIC 50–100 μg/mL, Tab. 1) on the *Escherichia coli* strain (lower than Nitrofural (MIC 1.5 μg/mL), similar to Trimetoprim (MIC 50 μg/mL)).

It is important to note that potassium thiolates **2.1–2.26**, as more water-soluble in most cases, are less active (MIC 100 μg/mL, Table 2). The antimicrobial activity of compounds **2.1–2.26** and **3.1–3.26** towards the strain of *Pseudomonas aeruginosa* is also moderate (MIC 50–100 μg/mL) and substantially inferior to the inhibitory action of Nitrofural (MIC 6.25 μg/mL, tables 1 and 2).

**Tab. 2.** Antimicrobial activities 8-R^1^-9-R^2^-10-R^3^-3-R-6-thio-6,7-dihydro-2H-[1, 2, 4]triazino[2, 3-*c*]quinazoline-2-ones (**3.1–3.26**)

Comp.	R	R^1^ = R^2^ = R^3^ = H (if not specified)	Investigated strains							
			*E. coli*		*S. aureus*		*P. aeruginosa*		*C. albicans*	
			MIC μg/mL	MBC μg/mL	MIC μg/mL	MBC μg/mL	MIC μg/mL	MBC μg/mL	MIC μg/mL	MBC μg/mL
3.1	CH_3_		100	100	100	200	50	200	100	100
3.2	C_6_H_5_		50	100	12.5	50	100	100	100	100
3.3	C_6_H_4_(CH_3_)-*p*		50	50	12.5	100	50	100	50	50
3.4	C_6_H_4_(CH_3_)_2_-*3,4*		50	100	100	200	50	100	50	100
3.5	C_6_H_4_(C_2_H_5_)-*p*		50	50	12.5	25	50	100	100	100
3.6	C_6_H_4_(*i*-C_3_H_7_)-*p*		50	100	12.5	50	50	100	100	100
3.7	C_6_H_4_(*tert*-C_4_H_9_)-*p*		50	100	6.25	12.5	50	200	50	100
3.9	C_6_H_4_(OC_2_H_5_)-*p*		100	100	25	100	50	100	100	100
3.10	C_6_H_4_F-*p*		50	100	12.5	50	50	200	50	100
3.11	C_6_H_5_	R^1^ = CH_3_	50	100	25	50	100	100	50	100
2.11	C_6_H_5_	R^3^= F	100	200	6.25	50	100	200	50	50
2.15	C_6_H_5_	R^3^= F	100	200	6.25	50	100	200	50	50
3.16	C_6_H_5_(CH_3_)-*p*	R^3^ = Cl	100	200	12.5	100	100	200	50	100
3.18	C_6_H_4_F-*p*	R^3^ = Cl	100	200	25	25	50	100	100	100
3.19	C_6_H_4_F-*p*	R^1^ = Br	50	100	100	200	100	100	100	100
3.20	C_6_H_4_F-*p*	R^2^ = Br	100	200	25	50	50	200	50	50
3.21	C_6_H_5_	R^3^ = Br	100	200	25	25	100	200	50	50
3.22	C_6_H_4_(CH_3_)-*p*	R^3^ = Br	50	100	50	200	100	200	50	50
3.23	C_6_H_4_F-*p*	R^3^ = Br	100	200	12.5	50	50	200	50	50
3.24	C_6_H_4_(OCH_3_)-*p*	R^3^ = Br	100	200	12.5	200	100	200	50	50
Trimethoprim		–	50	50	31.2	62,5	62.5	125	62.5	125
Nitrofural		–	1,5	–	6.25	–	6.25	–	25.0	–

At the same time, compounds **3.1–3.26** revealed high activity against *Staphylococcus aureus* (MIC 6.25–100 μg/mL), comparable to Nitrofural (MIC 6.25 μg/mL, Table 2). For potassium thiolates **2.1–2.26**, increasing antimicrobial activity was observed. It is important to note that the inhibitory action in lower concentrations was higher for derivatives with halogen substituents in the quinazoline cycle. Compounds **2.1–2.26** and **3.1–3.26** moderately inhibited *Candida albicans* (MIC 50-100 μg/mL), and revealed antifungal action comparable to Nitrofural (MIC 25 μg/mL, tables 1 and 2). Analysis of the structure–antimicrobial activity relationships showed the absence of obvious correlations. Antimicrobial activities, within the scope of each strain, were on similar levels.

## Experimental

### Chemistry

#### General Methods

Melting points were determined in open capillary tubes and were uncorrected. The elemental analyses (C, H, N, S) were performed using the ELEMENTAR vario EL Cube analyzer (USA). Analyses were indicated by the symbols of the elements or functions within ±0.3% of the theoretical values. The IR spectra (4000–600 cm^-1^) were recorded on a Bruker ALPHA FT-IR spectrometer (Bruker Bioscience, Germany) using a module for measuring attenuated total reflection (ATR). The ^1^H NMR spectra (400 MHz) and ^13^C NMR spectra (100 MHz) were recorded on a Varian-Mercury 400 (Varian Inc., Palo Alto, CA, USA) spectrometer with TMS as the internal standard in DMSO-cf_6_ solution. The LC-MS were recorded using a chromato-mass spectrometric system which consisted of a highperformance liquid chromatograph «Agilent 1100 Series» (Agilent, Palo Alto, CA, USA) equipped with a diode-matrix and mass-selective detector «Agilent LC/MSD SL» (atmospheric pressure chemical ionization – APCI). The electron impact mass spectra (EI-MS) were recorded on a Varian 1200 L instrument at 70 eV (Varian, USA). The purity of all obtained compounds was checked by ^1^H-NMR and LC-MS.

Substances **1.1–1.26** were synthesized according to the reported procedures [14]. Other starting materials and solvents were obtained from commercially available sources and used without additional purification.

### General procedures for the synthesis of potassium 8-R^1^-9-R^2^-10-R^3^-3-R-2-oxo-2H-[1, 2, 4]triazino[2, 3-c]quinazolin-6-thiolates (2.1-2.26) were:

#### Method A

0.76 g (10 mmol) of carbon disulfide was added with stirring to a solution of 0.56 g (10 mmol) of potassium hydroxide in 20 mL of ethanol. Proper 6-R-3-(3-R^1^-4-R^2^-5-R^3^-2-aminophenyl)-2*H*-[1, 2, 4]triazin-5-one (**1**) (10 mmol) was added to the obtained solution and refluxed for 4-10 hours. The resulting mixture was cooled, and the solid was filtered and dried.

#### Method B

1.60 g (10 mmol) of potassium *O*-ethyl dithiocarbonate was added to the suspension of proper 6-R-3-(3-R^1^-4-R^2^-5-R^3^-2-aminophenyl)-2*H*-[1, 2, 4]triazin-5-one (**1**) (10 mmol) in 20 mL of 2-propanol and refluxed for 4–10 hours. The resulting mixture was cooled, and the solid was filtered and dried.

#### Potassium 3-methyl-2-oxo-2H-[1, 2, 4]triazino[2, 3-c]quinazoline-6-thiolate (2.1)

Yield: 73.5% (Method A), 84% (Method B), mp >320°C; IR (cm^-1^): 3407, 3290, 3063, 2984, 2909, 2842, 1621, 1566, 1520, 1472, 1430, 1379, 1337, 1298, 1264, 1239, 1210, 1166, 1150, 1030, 944, 862, 766, 735, 688, 661, 636, 614; Anal. Calcd for C_11_H_7_N_4_OSK: C, 46.79; H, 2.50; N, 19.84; S, 11.35; Found: C, 46.78; H, 2.50; N, 19.83; S, 11.35.

#### Potassium 2-oxo-3-phenyl-2H-[1, 2, 4]triazino[2, 3-c]quinazoline-6-thiolate (2.2)

Yield: 75% (Method A), 93% (Method B), mp >320°C; IR (cm^-1^): 1620, 1602, 1570, 1524, 1493, 1474, 1463, 1432, 1371, 1345, 1319, 1296, 1277, 1253, 1232, 1171, 1155, 1077, 1034, 1001, 985, 939, 856, 818, 755, 695, 656, 606; Anal. Calcd for C_16_H_9_N_4_OSK: C, 55.79; H, 2.63; N, 16.27; S, 9.31; Found: C, 55.78; H, 2.65; N, 16.27; S, 9.31.

#### Potassium 3-(4-methylphenyl)-2-oxo-2H-[1, 2, 4]triazino[2, 3-c]quinazoline-6-thiolate (2.3)

Yield: 77% (Method A), 77% (Method B), mp >320°C; IR (cm^-1^): 3079, 1620, 1602, 1570, 1524, 1477, 1463, 1433, 1405, 1371, 1346, 1320, 1300, 1278, 1246, 1233, 1172, 1112, 1075, 1035, 1023, 985, 939, 874, 855, 835, 798, 783, 755, 715, 695, 684, 660, 641, 629, 611; Anal. Calcd for C_17_H_11_N_4_OSK: C, 56.96; H, 3.09; N, 15.64; S, 8.95; Found: C, 56.94; H, 3.09; N, 15.65; S, 8.94.

#### Potassium 3-(3,4-dimethylphenyl)-2-oxo-2H-[1, 2, 4]triazino[2,3-c]quinazoline-6-thiolate (2.4)

Yield: 67.2% (Method A), 92.1% (Method B), mp >320°C; IR (cm^-1^): 3438, 3394, 3292, 3054, 3021, 2962, 2916, 1660, 1644, 1626, 1602, 1568, 1537, 1524, 1475, 1432, 1393, 1369, 1347, 1295, 1274, 1254, 1232, 1185, 1167, 1126, 1078, 1013, 982, 950, 903, 890, 868, 849, 833, 756, 736, 713, 704, 688, 659, 634; Anal. Calcd for C_18_H_14_N_4_OSK: C, 58.04; H, 3.52; N, 15.04; S, 8.61; Found: C, 58.06; H, 3.58; N, 15.02; S, 8.59.

#### Potassium 3-(4-ethylphenyl)-2-oxo-2H-[1, 2, 4]triazino[2, 3-c]quinazoline-6-thiolate (2.5)

Yield: 79% (Method B), mp >320°C; IR (cm^-1^): 3389, 3238, 2955, 2927, 1620, 1602, 1571, 1520, 1478, 1464, 1435, 1412, 1370, 1348, 1318, 1303, 1280, 1254, 1233, 1175, 1161, 1120, 1075, 1045, 1019, 984, 940, 881, 846, 761, 735, 697, 688, 674, 657, 641, 630, 610; Anal. calcd. for C_18_H_13_N_4_OSK: C, 58.04; H, 3.52; N, 15.04; S, 8.61; Found: C, 58.06; H, 3.51; N, 15.05; S, 8.60.

#### Potassium 3-(4-isopropylphenyl)-2-oxo-2H-[1, 2, 4]triazino[2, 3-c]quinazoline-6-thiolate (2.6)

Yield: 90% (Method B), mp >320°C; IR (cm^-1^): 3411, 3056, 2954, 2926, 2867, 1621, 1604, 1570, 1538, 1505, 1477, 1463, 1440, 1415, 1369, 1343, 1302, 1279, 1245, 1234, 1174, 1117, 1074, 1051, 986, 942, 870, 847, 780, 754, 694, 686, 674, 658, 643, 627, 612; Anal. calcd. for C_19_H_15_N_4_OSK: C, 59.04; H, 3.91; N, 14.50; S, 8.30; Found: C, 59.05; H, 3.90; N, 14.52; S, 8.28.

#### Potassium 3-(4-tert-butylphenyl)-2-oxo-2H-[1, 2, 4]triazino[2, 3-c]quinazoline-6-thiolate (2.7)

Yield: 67% (Method A), 78% (Method B), mp >320°C; IR (cm^-1^): 3563, 3405, 2963, 2867, 1627, 1607, 1574, 1539, 1509, 1478, 1444, 1412, 1345, 1304, 1280, 1269, 1255, 1233, 1198, 1179, 1135, 1114, 1075, 986, 943, 847, 756, 694, 685, 662, 611; Anal. calcd. for C_20_H_17_N_4_OSK: C, 59.97; H, 4.28; N, 13.99; S, 8.01; Found: C, 59.95; H, 4.28; N, 13.99; S, 8.03.

#### Potassium 3-(4-methoxyphenyl)-2-oxo-2H-[1, 2, 4]triazino[2, 3-c]quinazoline-6-thiolate (2.8)

Yield: 83% (Method A), 99% (Method B), mp >320°C; IR (cm^-1^): 2963, 2904, 2830, 1650, 1601, 1570, 1536, 1505, 1477, 1439, 1417, 1368, 1343, 1315, 1297, 1280, 1261, 1254, 1232, 1168, 1133, 1075, 1029, 1018, 1008, 984, 939, 857, 837, 819, 800, 754, 724, 705, 689, 657, 635, 625, 611; Anal. Calcd for C_17_H_11_N_4_O_2_SK: C, 54.53; H, 2.96; N, 14.96; S, 8.56; Found: C, 54.53; H, 2.97; N, 14.95; S, 8.55.

#### Potassium 3-(4-ethoxyphenyl)-2-oxo-2H-[1, 2, 4]triazino[2, 3-c]quinazoline-2-thiolate (2.9)

Yield: 91% (Method B), mp >320°C; IR (cm^-1^): 2980, 1636, 1602, 1571, 1532, 1512, 1477, 1435, 1418, 1368, 1344, 1298, 1233, 1172, 1155, 1123, 1075, 1039, 986, 942, 921, 896, 835, 825, 799, 781, 762, 722, 704, 692, 672, 645, 625; Anal. calcd. for C_18_H_13_N_4_02SK: C, 55.65; H, 3.37; N, 14.42; S, 8.25; Found: C, 55.67; H, 3.36; N, 14.42; S, 8.24.

#### Potassium 3-(4-fluorophenyl)-2-oxo-2H-[1, 2, 4]triazino[2, 3-c]quinazoline-6-thiolate (2.10)

Yield: 86% (Method A), 94% (Method B), mp >320°C; IR (cm^-1^): 3352, 2965, 1635, 1599, 1572, 1532, 1505, 1474, 1435, 1408, 1370, 1344, 1294, 1280, 1231, 1173, 1153, 1131, 1095, 1071, 1015, 982, 954, 939, 844, 818, 801, 783, 767, 719, 695, 659, 635, 626, 611; Anal. calcd. for C_16_H_8_FN_4_OSK: C, 53.02; H, 2.22; N, 15.46; S, 8.85; Found: C, 53.05; H, 2.21; N, 15.45; S, 8.84.

#### Potassium 8-methyl-2-oxo-3-phenyl-2H-[1, 2, 4]triazino[2, 3-c]quinazoline-6-thiolate (2.11)

Yield: 91% (Method A), 94% (Method B), mp >320°C; IR (cm^-1^): 3350, 3049, 2965, 2915, 1636, 1614, 1599, 1575, 1532, 1480, 1443, 1412, 1364, 1350, 1291, 1260, 1241, 1192, 1179, 1132, 1097, 1023, 1001, 975, 833, 813, 750, 706, 686, 658, 619; Anal. calcd. for C_17_H_11_N_4_OSK: C, 56.96; H, 3.09; N, 15.63; S, 8.95; Found: C, 56.97; H, 3.09; N, 15.64; S, 8.95.

#### Potassium 9-fluoro-2-oxo-3-phenyl-2H-[1, 2, 4]triazino[2, 3-c]quinazoline-6-thiolate (2.12)

Yield: 81% (Method B); mp >320°C; IR (cm^-1^): 3187, 3124, 3079, 3059, 3010, 2967, 2927, 1665, 1618, 1556, 1516, 1490, 1444, 1393, 1355, 1315, 1295, 1276, 1261, 1190, 1171, 1143, 1104, 1085, 1034, 1018, 1001, 977, 939, 860, 831, 814, 788, 776, 747, 709, 681, 660, 638, 618; Anal. calcd. for C_16_H_8_FN_4_OSK: C, 53.02; H, 2.22; N, 15.46; S, 8.85; Found: C, 53.06; H, 2.22; N, 15.44; S, 8.83.

#### Potassium 9-fluoro-3-(4-fluorophenyl)-2-oxo-2H-[1, 2, 4]triazino[2, 3-c]quinazoline-6-thiolate (2.13)

Yield: 83% (Method A), 87% (Method B); mp >320°C; IR (cm^-1^): 3099, 3058, 3014, 2961, 2925, 1650, 1625, 1599, 1557, 1518, 1503, 1433, 1413, 1392, 1354, 1294, 1273, 1237, 1184, 1166, 1143, 1103, 1017, 977, 943, 867, 841, 826, 804, 772, 752, 718, 702, 682, 672, 640, 620; Anal. calcd. for C_16_H_7_F_2_N_4_OSK: C, 50.52; H, 1.85; N, 14.73; S, 8.43; Found: C, 50.56; H, 1.85; N, 14.72; S, 8.41

#### Potassium 9-fluoro-3-(4-methoxyphenyl)-2-oxo-2H-[1, 2, 4]triazino[2, 3-c]quinazoline-6-thiolate (2.14)

Yield: 83% (Method B); mp >320°C; IR (cm^-1^): 3082, 3010, 2963, 2879, 2838, 2774, 1658, 1600, 1574, 1552, 1538, 1504, 1487, 1435, 1398, 1354, 1304, 1267, 1188, 1168, 1107, 1081, 1027, 1014, 976, 938, 862, 837, 808, 798, 776, 765, 750, 708, 676, 641, 617; Anal. calcd. for C_17_H_10_FN_4_O_2_SK: C, 52.03; H, 2.57; N, 14.28; S, 8.17; Found: C, 52.02; H, 2.56; N, 14.29; S, 8.18.

#### Potassium 10-chloro-2-oxo-3-phenyl-2H-[1, 2, 4]triazino[2, 3-c]quinazoline-6-thiolate (2.15)

Yield: 75% (Method A), 82% (Method B), mp >320°C; IR (cm^-1^): 3899, 3851, 3645, 3626, 3389, 1640, 1625, 1599, 1558, 1538, 1493, 1474, 1449, 1410, 1368, 1346, 1319, 1278, 1251, 1237, 1174, 1130, 1094, 1038, 1004, 991, 959, 885, 863, 833, 818, 756, 700, 688, 674, 652, 624; Anal. calcd. for C_16_H_8_CIN_4_OSK: C, 50.72; H, 2.13; N, 14.79; S, 8.46; Found: C, 50.74; H, 2.13; N, 14.78; S, 8.45.

#### Potassium 10-chloro-3-(4-methylphenyl)-2-oxo-2H-[1, 2, 4]triazino[2, 3-c]quinazoline-6-thiolate (2.16)

Yield: 90% (Method B); mp >320°C; IR (cm^-1^): 1624, 1556, 1537, 1475, 1447, 1407, 1368, 1342, 1278, 1237, 1186, 1174, 1133, 1091, 1059, 1021, 994, 959, 882, 864, 836, 828, 770, 746, 713, 700, 673, 656, 642, 631; Anal. calcd. for C_17_H_10_CIKN_4_OS: C, 51.97; H, 2.57; N, 14.26; S, 8.16; Found: C, 51.94; H, 2.57; N, 14.28; S, 8.18.

#### Potassium 10-chloro-3-(4-methoxyphenyl)-2-oxo-2H-[1, 2, 4]triazino[2, 3-c]quinazoline-6-thiolate (2.17)

Yield: 82% (Method B); mp >320°C; IR (cm^-1^): 3056, 2981, 2942, 2915, 1649, 1608, 1596, 1574, 1552, 1541, 1521, 1503, 1478, 1456, 1428, 1394, 1344, 1305, 1271, 1237, 1188, 1175, 1150, 1124, 1114, 1096, 1017, 966, 902, 873, 839, 822, 769, 760, 728, 698, 673, 629; Anal. calcd. for C_17_H_10_CIN_4_O_2_SK: C, 49.93; H, 2.46; N, 13.70; S, 7.84; Found: C, 49.97; H, 2.46; N, 13.68; S, 7.82.

#### Potassium 10-chloro-3-(4-fluorophenyl)-2-oxo-2H-[1, 2, 4]triazino[2, 3-c]quinazoline-6-thiolate (2.18)

Yield: 66% (Method A), 79% (Method B), mp >320°C; IR (cm^-1^): 1628, 1598, 1557, 1539, 1504, 1474, 1446, 1403, 1367, 1341, 1320, 1299, 1278, 1237, 1172, 1157, 1130, 1100, 1089, 1065, 1009, 994, 958, 884, 867, 847, 831, 771, 747, 717, 700, 673, 657, 639, 627; Anal. calcd. for C_16_H_7_CIFKN_4_OS: C, 48.42; H, 1.78; N, 14.12; S, 8.08; Found: C, 48.46; H, 1.78; N, 14.13; S, 8.10.

#### Potassium 8-bromo-3-(4-fluorophenyl)-2-oxo-2H-[1, 2, 4]triazino[2, 3-c]quinazoline-6-thiolate (2.19)

Yield: 86% (Method B), mp >320°C; IR (cm^-1^): 2954, 1621, 1593, 1556, 1532, 1504, 1472, 1446, 1411, 1383, 1365, 1345, 1311, 1285, 1271, 1250, 1233, 1218, 1160, 1147, 1112, 1074, 991, 964, 922, 847, 817, 751, 717, 707, 692, 664, 627, 609; Anal. calcd. for C_16_H_7_BrFN_4_OSK: C, 43.54; H, 1.60; N, 12.70; S, 7.27; Found: C, 43.52; H, 1.61; N, 12.71; S, 7.28.

#### Potassium 9-bromo-3-(4-fluorophenyl)-2-oxo-2H-[1, 2, 4]triazino[2, 3-c]quinazoline-6-thiolate (2.20)

Yield: 99% (Method B); mp >320°C; IR (cm^-1^): 3390, 1632, 1591, 1557, 1538, 1505, 1455, 1406, 1372, 1337, 1288, 1263, 1232, 1159, 1102, 1070, 990, 941, 894, 845, 818, 763, 715, 669, 655, 621; Anal. calcd. for C_16_H_7_BrFN_4_OSK: C, 43.54; H, 1.60; N, 12.70; S, 7.27; Found: C, 43.50; H, 1.60; N, 12.72; S, 7.22.

#### Potassium 10-bromo-2-oxo-3-phenyl-2H-[1, 2, 4]triazino[2, 3-c]quinazoline-6-thiolate (2.21)

Yield: 65% (Method B); mp >320°C; IR (cm^-1^): 3149, 3097, 3057, 2979, 2909, 1657, 1630, 1614, 1595, 1555, 1507, 1474, 1445, 1425, 1393, 1342, 1283, 1260, 1232, 1190, 1152, 1120, 1088, 1034, 1014, 957, 922, 907, 869, 837, 813, 794, 751, 735, 688, 669, 645, 624; Anal. calcd. for C_16_H_8_BrN_4_OSK: C, 45.40; H, 1.90; N, 13.23; S, 7.57; Found: C, 45.44; H, 1.90; N, 13.22; S, 7.55.

#### Potassium 10-bromo-3-(4-methylphenyl)-2-oxo-2H-[1, 2, 4]triazino[2, 3-c]quinazoline-6-thiolate (2.22)

Yield: 91% (Method B); mp >320°C; IR (cm^-1^): 1623, 1597, 1556, 1537, 1472, 1444, 1404 1369, 1339, 1322, 1276, 1242, 1185, 1173, 1134, 1080, 1021, 993, 953, 881, 860, 836 826, 800, 769, 730, 713, 699, 659, 630; Anal. calcd. for C_17_H_10_BrN_4_OSK: C, 46.69; H 2.30; N, 12.81; S, 7.33; Found: C, 46.65; H, 2.30; N, 12.83; S, 7.35.

#### Potassium 10-bromo-3-(4-fluorophenyl)-2-oxo-2H-[1, 2, 4]triazino[2, 3-c]quinazoline-6-thiolate (2.23)

Yield: 88% (Method B); mp >320°C; IR (cm^-1^): 1627, 1596, 1556, 1538, 1504, 1471, 1444 1401, 1369, 1339, 1277, 1241, 1173, 1158, 1132, 1101, 1077, 1009, 993, 953, 882, 846 830, 771, 732, 717, 700, 673, 660, 638, 626; Anal. calcd. for C_16_H_7_BrFN_4_OSK: C, 43.54 H, 1.60; N, 12.70; S, 7.27; Found: C, 43.58; H, 1.60; N, 12.68; S, 7.25.

#### Potassium 10-bromo-3-(4-methoxyphenyl)-2-oxo-2H-[1, 2, 4]triazino[2, 3-c]quinazoline-6-thiolate (2.24)

Yield: 89% (Method B); mp >320°C; IR (cm^-1^): 3079, 3050, 3018, 2988, 2930, 2833, 1622 1606, 1571, 1555, 1533, 1511, 1472, 1444, 1434, 1409, 1371, 1339, 1306, 1284, 1271 1242, 1173, 1133, 1123, 1112, 1079, 1030, 1009, 992, 953, 862, 837, 830, 805, 769, 730 702, 673, 659, 628; Anal. calcd. for C_17_H_10_BrN_4_O_2_SK: C, 45.04; H, 2.22; N, 12.36; S, 7.07 Found: C, 45.00; H, 2.22; N, 12.37; S, 7.10

#### Potassium 3-(4-fluorophenyl)-10-iodo-2-oxo-2H-[1, 2, 4]triazino[2, 3-c]quinazoline-6-thiolate (2.25)

Yield: 89% (Method B); mp >320°C; IR (cm^-1^): 3499, 3359, 1678, 1628, 1609, 1590, 1555 1538, 1521, 1505, 1487, 1472, 1444, 1402, 1374, 1337, 1327, 1296, 1276, 1241, 1215 1172, 1158, 1126, 1100, 1074, 1011, 992, 950, 875, 844, 831, 824, 815, 772, 759, 747 718, 700, 687, 672, 648, 637, 621; Anal. calcd. for C_16_H_7_FIN_4_OSK: C, 39.35; H, 1.44; N 11.47; S, 6.57; Found: C, 39.39; H, 1.44; N, 11.45; S, 6.56.

#### Potassium 10-iodo-3-(4-methoxyphenyl)-2-oxo-2H-[1, 2, 4]triazino[2, 3-c]quinazoline-6-thiolate (2.26)

Yield: 90% (Method B); mp >320°C; IR (cm^-1^): 3080, 3051, 3020, 2971, 2930, 2834, 1622 1606, 1590, 1571, 1552, 1532, 1511, 1472, 1445, 1435, 1421, 1405, 1368, 1338, 1306 1284, 1270, 1243, 1173, 1139, 1124, 1112, 1078, 1030, 1010, 992, 951, 880, 861, 837 830, 805, 769, 723, 702, 652, 628; Anal. calcd. for C_17_H_10_IN_4_O_2_SK: C, 40.81; H, 2.01; N 11.20; S, 6.41; Found: C, 40.84; H, 2.01; N, 11.18; S, 6.40.

### The general procedure for the synthesis of 8-R^1^-9-R^2^-10-R^3^-3-R-6-thio-6,7-dihydro-2H-[1, 2, 4]triazino[2, 3-c]quinazolin-2-ones (3.1–3.26) was the following:

Proper potassium 8-R^1^-9-R^2^-10-R^3^-3-R-2-oxo-2H-[1, 2, 4]triazino[2, 3-*c*]quinazolin-2-one (**2**) (10 mmol) was dissolved in 20 mL of water and acidified by the addition of hydrochlori acid to pH 2–3. The formed precipitate was filtered and dried.

#### 3-Methyl-6-thioxo-6,7-dihydro-2H-[1, 2, 4]triazino[2, 3-c]quinazolin-2-one (3.1)

Yield: 99%, mp 254-256°C; IR (CM^-1^): 3118, 3056, 3029, 2954, 2917, 2849, 1767, 1743 1687, 1637, 1616, 1594, 1564, 1519, 1471, 1455, 1421, 1381, 1360, 1322, 1303, 1277 1257, 1225, 1169, 1128, 1111, 1039, 1025, 988, 949, 862, 771, 752, 716, 671, 655, 623 ^1^H NMR: δ=2.34 (s, 3H, CH3), 7.48-7.43 (m, 2H, H-8, 10), 7.82 (t, 1H, J^3^ = 7.9, J4 = 1.4, H-9), 8.29 (d, 1H, J = 7.9, H-11), 13.83 (s, 1H, NH); ^13^C NMR: δ =20.04 (CH_3_), 118.29 (11a), 127.49 (8), 130.30 (10), 136.24 (11), 141.07 (9), 144.04 (3), 150.25 (11b), 154.72 (7a), 160.16 (2), 170,1 (6); EI-MS, m/z (Irel, %) = 246 (5.8), 245 (11.4), 244 (M+», 65.5), 205 (2.1), 204 (13.4), 203 (100.0), 198 (10.2), 174 (10.2), 171 (7.4), 170 (12.4), 163 (4.0), 161 (35.4), 160 (6.7), 146 (2.8), 145 (76.7), 144 (21.1), 143 (22.5), 142 (5.8), 134 (13.6), 117 (8.6), 116 (9.0), 108 (6.9), 107 (7.7), 105 (8.8), 103 (11.3), 102 (35.9), 91 (6.1), 90 (42.7), 89 (5.3), 88 (5.1), 86 (11.1), 78 (5.0), 77 (8.9), 76 (15.5), 75 (23.4), 74 (5.1), 70 (10.7), 69 (10.5), 65 (8.5), 64 (27.5), 63 (19.8), 62 (5.9); LC-MS, m/z = 245 [M+1], 247 [M+3]; Anal. Calcd for C_11_H_8_N_4_OS: C, 54.09; H, 3.30; N, 22.94; S, 13.13; Found: C, 54.07; H, 3.31; N, 22.93; S, 13.14.

#### 3-Phenyl-6-thioxo-6, 7-dihydro-2H-[1, 2, 4]triazino[2, 3-c]quinazolin-2-one (3.2)

Yield: 96%, mp >300°C; IR (cm^-1^): 3358, 3182, 3014, 1654, 1608, 1589, 1546, 1512, 1497, 1456, 1398, 1356, 1337, 1302, 1278, 1243, 1205, 1178, 1156, 1128, 1091, 1046, 1024, 981, 943, 869, 833, 810, 776, 753, 721, 686, 632; ^1^H NMR: δ=7.61-7.42 (m, 5H, H-3', 4', 5', 8, 10), 7.82 (t, 1H, J^3^ = 7.9, J^4^ = 1.4, H-9), 8.36-8.20 (m, 3H, H-2', 6', 11,), 13.92 (s, 1H, NH); EI-MS, m/z (Irel, %) = 308 (7.2), 307 (25.8), 306 (M+, 69.9), 229 (5.1), 205 (35.2), 204 (74.2), 203 (96.7), 187 (11.1), 176 (5.8), 175 (6.3), 174 (19.1), 171 (9.6), 170 (59.5), 163 (6.4), 162 (13.9), 161 (100.0), 160 (36.6), 159 (7.7), 146 (27.2), 145 (98.6), 144 (43.2), 143 (82.0), 142 (13.8), 135 (8.0), 134 (54.3), 129 (7.3), 122 (7.3), 118 (8.0), 117 (49.2), 116 (13.9), 108 (5.0), 107 (5.4), 104 (7.4), 103 (37.1), 102 (68.7), 91 (6.8), 90 (56.3), 89 (23.1), 88 (5.7), 86 (7.1), 77 (21.6), 76 (35.4), 75 (24.1), 69 (5.9), 64 (13.2), 63 (28.6), 62 (8.6), 52 (8.3), 51 (18.2), 50 (14.9); LC-MS, m/z = 307 [M+1]; Anal. Calcd for C_16_H_10_N_4_OS: C, 62.73; H, 3.29; N, 18.29; S, 10.47; Found: C, 62.70; H, 3.30; N, 18.27; S, 10.46.

#### 3-(4-Methylphenyl)-6-thioxo-6, 7-dihydro-2H-[1, 2, 4]triazino[2, 3-c]quinazolin-2-one (3.3)

Yield: 86%, mp >300°C; IR (cm^-1^): 3560, 3171, 3112, 3065, 3014, 2975, 2927, 1647, 1618, 1603, 1573, 1548, 1518, 1506, 1483, 1454, 1395, 1364, 1345, 1307, 1268, 1248, 1195, 1182, 1160, 1149, 1108, 1081, 1026, 1014, 961, 943, 886, 869, 833, 810, 776, 753, 721, 686, 632,616; ^1^H NMR: δ=2.39 (s, 3H, CH3), 7.35 (d, 2H, J = 8.2, H-3', 5'), 7.52-7.43 (m, 2H, H-8, 10), 7.82 (t, 1H, J^3^ = 7.9, J^4^ = 1.4, H-9), 8.24 (d, 2H, J = 8.2, H-2‗, 6‗), 8.32 (d, 1H, J = 7.9, H-11), 13.88 (s, 1H, NH); ^13^C NMR: δ=21.50 (CH3), 110.01 (11a), 115.92 (8), 117.69 (10), 128.79 (2',6'-Ph), 128.93 (11), 129.06 (9, 1'-Ph), 129.19 (3',5'-Ph), 130.73 (3), 133.53 (4'-Ph), 140.24 (11-b), 150.20 (7a), 158.68 (2), 168.79 (6); EI-MS, m/z (Irel, %) = 320 (M+», 4.1), 205 (6.1), 204 (12.9), 203 (100.0), 171 (8.2), 170 (10.8), 163 (3.3), 161 (24.3), 160 (8.8), 149 (15.0), 146 (6.4), 145 (69.1), 144 (11.4), 143 (22.4), 134 (16.0), 129 (8.3), 119 (6.7), 118 (9.8), 117 (49.6), 116 (32.2), 103 (8.1), 102 (22.1), 97 (7.0), 91 (9.4), 90 (29.1), 89 (14.1), 85 (6.3), 83 (10.0), 77 (8.4), 76 (7.7), 75 (7.4), 73 (5.2), 71 (6.0), 69 (8.7), 64 (5.6), 63 (8.9), 60 (6.5), 57 (14.6), 56 (7.0), 55 (12.8), 51 (7.9), 50 (5.3), 45 (7.9), 43 (14.9), 41 (14.0); LC-MS, m/z = 321 [M+1], 322 [M+2]; Anal. Calcd for C_17_H_12_N_4_OS: C, 63.73; H, 3.78; N, 17.49; S, 10.01; Found: C, 63.74; H, 3.79; N, 17.48; S, 10.03.

#### 3-(3,4-Dimethylphenyl)-6-thioxo-6, 7-dihydro-2H-[1, 2, 4]triazino[2, 3-c]quinazolin-2-one (3.4)

Yield: 93%, mp >310°C; IR (cm^-1^): 3246, 3192, 3119, 3070, 3033, 2969, 2923, 1674, 1656, 1618, 1551, 1535, 1516, 1499, 1483, 1447, 1389, 1343, 1305, 1258, 1220, 1188, 1144, 1131, 1107, 1083, 1030, 993, 958, 906, 868, 854, 834, 773, 756, 747, 711, 692, 682, 618; 1H NMR: δ=2.28 (s, 6H, 3,4-(CH_3_)_2_), 7.27 (d, 1H, J = 8.1, H-5'), 7.44 (m, 2H, H-8, 10), 7.81 (t, 1H, J^3^ = 7.9, J^4^ = 1.4, H-9), 8.08 (m, 2H, H-6‗, 2'), 8.29 (d, 1H, J = 7.9, H-11), 13.9 (s, 1H, NH); ^13^C NMR: δ=20.00 (3-CH_3_), 20.15 (4-CH3), 115.83 (11a), 116.15 (8), 125.80 (5'-Ph), 126.73 (10), 127.38 (11), 129.95 (6'-Ph), 130.05 (3), 130.52 (9), 136.22 (2'-Ph), 136.57 (3'-Ph), 137.87 (1'-Ph), 140.55 (4'-Ph), 149.37 (11-b), 151.02 (7a), 159.98 (2), 171.05 (6); EI-MS, m/z (Irel, %) = 334 (M+», 2.8), 205 (5.7), 204 (12.0), 203 (100.0), 171 (9.4), 170 (10.2), 163 (2.1), 161 (26.5), 160 (7.2), 149 (18.0), 146 (9.1), 145 (77.9), 144 (14.5), 143 (28.3), 134 (19.7), 132 (7.3), 131 (41.0), 130 (19.7), 129 (14.9), 123 (6.6), 119 (7.0), 118 (10.4), 117 (33.3), 116 (93.0), 115 (11.5), 105 (5.9), 104 (7.9), 103 (22.3), 102 (25.2), 97 (9.3), 91 (8.7), 90 (19.5), 89 (15.3), 85 (5.8), 84 (5.1), 83 (11.3), 77 (16.1), 76 (10.5), 75 (9.9), 74 (5.2), 73 (13.1), 69 (6.9), 64 (7.0), 63 (10.9), 60 (13.5), 57 (22.2), 56 (8.0), 55 (16.1), 51 (11.4), 50 (6.1), 45 (18.7), 44 (8.4), 43 (23.7); LC-MS, m/z = 335 [M+1], 337 [M+3]; Anal. Calcd for C_18_H_14_N_4_OS: C, 64.65; H, 4.22; N, 16.75; S, 9.59; Found: C, 64.67; H, 4.21; N, 16.76; S, 9.61.

#### 3-(4-Ethylphenyl)-6-thioxo-6, 7-dihydro-2H-[1, 2, 4]triazino[2, 3-c]quinazolin-2-one (3.5)

Yield: 92%, mp >300°C; IR (cm^-1^): 3176, 3112, 3059, 3022, 2961, 2927, 1668, 1619, 1605, 1572, 1545, 1518, 1505, 1483, 1429, 1414, 1390, 1360, 1345, 1304, 1255, 1191, 1182, 1149, 1108, 1079, 1051, 1010, 976, 941, 868, 848, 834, 802, 748, 714, 701, 683, 629, 616; 1H NMR: δ=1.26 (t, J = 7.4 Hz, 3H, CH_2_CH_3_), 2.73 (q, J = 14.7, 7.4 Hz, 2H, CH_2_CH_3_), 7.28 (d, 2H, J = 8.2, H-3', 5'), 7.52-7.48 (m, 2H, H-8, 10), 7.82 (t, 1H, J = 7.8, H-9), 8.19 (d, 2H, J = 8.2, H-2‗, 6‗), 8.30 (d, 1H, J = 7.9, H-11), 13.90 (s, 1H, NH); LC-MS, m/z = 335 [M+1], 337 [M+3]; Anal. Calcd for C_18_H_14_N_4_OS: C, 64.65; H, 4.22; N, 16.75; S, 9.59; Found: C, 64.67; H, 4.22; N, 16.74; S, 9.58.

#### 3-(4-Isopropylphenyl)-6-thioxo-6,7-dihydro-2H-[1, 2, 4]triazino[2, 3-c]quinazolin-2-one (3.6)

Yield: 89%, mp >300°C; IR (cm^-1^): 3180, 3126, 3056, 3014, 2959, 2925, 2869, 1764, 1643, 1615, 1548, 1495, 1456, 1404, 1366, 1346, 1308, 1266, 1247, 1194, 1159, 1109, 1083, 1055, 1011, 946, 873, 846, 819, 793, 749, 694, 684, 628, 617; ^1^H NMR: δ= 1.25 (d, J = 6.7 Hz, 6H, -CH(CH_3_)_2_), 2.98 (quin, J = 6.7 Hz, 1H, -CH(CH_3_)_2_, 7.26 (d, 2H, J = 8.2, H-3', 5'), 7.50-7.45 (m, 2H, H-8, 10), 7.81 (t, 1H, J = 7.8, H-9), 8.17 (d, 2H, J = 8.2, H-2‗, 6‗), 8.28 (d, 1H, J = 7.7, H-11), 13.92 (s, 1H, NH); LC-MS, m/z = 349 [M +1], 351 [M +3]; Anal. calcd. for C_19_H_16_N_4_OS: C, 65.50; H, 4.63; N, 16.08; S, 9.20; Found: C, 65.51; H, 4.63; N, 16.07; S, 9.20.

#### 3-(4-tert-Butylphenyl)-6-thioxo-6, 7-dihydro-2H-[1, 2, 4]triazino[2, 3-c]quinazolin-2-one (3.7)

Yield: 91%, mp >300°C; IR (cm^-1^): 3201, 3126, 3077, 3061, 3016, 2961, 2933, 2903, 1766, 1643, 1617, 1549, 1513, 1496, 1485, 1405, 1370, 1345, 1307, 1266, 1250, 1192, 1158, 1147, 1124, 1107, 1083, 1011, 947, 875, 842, 798, 779, 751, 722, 687, 628, 616; LC-MS, m/z = 363 [M +1]; ^1^H NMR: δ= 1.39 (s, 9H, C(CH_3_)_3_), 7.17 (d, 2H, J = 8.2, H-3', 5'), 7.47-7.41 (m, 2H, H-8, 10), 7.77 (t, 1H, J = 7.8, H-9), 8.13 (d, 2H, J = 8.2, H-2‗, 6‗), 8.24 (d, 1H, J = 7.7, H-11), 13.81 (s, 1H, NH); Anal. calcd. for C_20_H_18_N_4_OS: C, 66.28; H, 5.01; N, 15.46; S, 8.85; Found: C, 66.29; H, 5.01; N, 15.45; S, 8.85.

#### 3-(4-Methoxyphenyl)-6-thioxo-6, 7-dihydro-2H-[1, 2, 4]triazino[2, 3-c]quinazolin-2-one (3.8)

Yield: 93%, mp 280-282°C; IR (cm^-1^): 3180, 3117, 3065, 3021, 2960, 2905, 2835, 1680, 1624, 1600, 1551, 1522, 1508, 1485, 1432, 1390, 1360, 1344, 1304, 1259, 1202, 1174, 1147, 1118, 1105, 1076, 1007, 941, 868, 851, 814, 786, 772, 752, 724, 706, 693, 680, 628, 614; ^1^H NMR: δ=3.83 (s, 3H, OCH_3_), 7.09 (d, 2H, J=8.8, H-3', 5'), 7.45 (m, 2H, H-8, 10), 7.81 (t, 1H, J3 = 7.9, J4 = 1.4, H-9), 8.33 (m, 3H, H-11, 2', 6'), 13.91 (s, 1H, NH); 13C NMR: δ =55.90 (OCH_3_), 114.35 (3',5'-Ph), 115.81 (11a), 116.15 (8), 124.76 (10), 125.80 (11), 126.67 (1'-Ph), 131.56 (2',6'-Ph), 136.17 (9), 137.84 (3), 148.57 (11-b), 150.88 (7a), 160.07 (2), 162.26 (4'-Ph), 171.03 (6); EI-MS, m/z (Irel, %) = 336 (M+, 7.1), 205 (5.6), 204 (11.9), 203 (100.0), 170 (9.5), 163 (3.4), 161 (25.0), 160 (7.7), 149 (6.5), 146 (6.9), 145 (69.7), 144 (11.2), 143 (24.3), 134 (21.2), 133 (47.1), 129 (7.7), 119 (11.3), 118 (7.1), 117 (19.5), 116 (5.5), 104 (6.3), 103 (20.3), 102 (20.4), 91 (6.1), 90 (29.9), 76 (9.0), 75 (6.6), 64 (7.2), 63 (8.8), 57 (5.3), 55 (6.4), 51 (5.1), 45 (8.9), 41 (6.2); LC-MS, m/z = 337 [M+1], 339 [M+3]; Anal. Calcd for C_17_H_12_N_4_O_2_S: C, 60.70; H, 3.60; N, 16.66; S, 9.53; Found: C, 60.69; H, 3.59; N, 16.64; S, 9.54.

#### 3-(4-Ethoxyphenyl)-6-thioxo-6, 7-dihydro-2H-[1, 2, 4]triazino[2, 3-c]quinazolin-2-one (3.9)

Yield: 91%, mp >300°C; IR (cm^-1^): 3177, 3114, 3061, 3016, 2979, 2926, 2901, 1659, 1618, 1601, 1546, 1504, 1484, 1421, 1395, 1365, 1344, 1308, 1261, 1241, 1190, 1175, 1150, 1121, 1109, 1088, 1034, 1014, 1004, 968, 944, 923, 898, 868, 843, 816, 799, 774, 753, 724, 685, 640, 627, 616; ^1^H NMR: 1.37 (t, J = 6.7 Hz, 3H, 0CH_2_CH_3_), 3.91 (quad, J = 6.7 Hz, 2H, -OCH_2_CH_3_), 7.01 (d, 2H, J=8.8, H-3', 5'), 7.38-7.46 (m, 2H, H-8, 10), 7.74 (t, 1H, J = 7.9, H-9), 8.27 (m, 3H, H-11, 2', 6'), 13.92 (s, 1H, NH); LC-MS, m/z = 351 [M+1]; Anal. calcd. for C_18_H_14_N_4_O_2_S: C, 61.70; H, 4.03; N, 15.99; S, 9.15; Found: C, 61.71; H, 4.03; N, 15.98; S, 9.15.

#### 3-(4-Fluorophenyl)-6-thioxo-6, 7-dihydro-2H-[1, 2, 4]triazino[2, 3-c]quinazolin-2-one (3.10)

Yield: 92%, mp >300°C; IR (cm^-1^): 3564, 3487, 3111, 3070, 3014, 2926, 2889, 1755, 1656, 1620, 1601, 1549, 1520, 1504, 1484, 1398, 1368, 1345, 1310, 1295, 1267, 1250, 1226, 1196, 1160, 1103, 1013, 963, 944, 894, 853, 824, 805, 779, 754, 723, 702, 686, 633, 616; ^1^H NMR: δ=7.27 (t, J=8.0, 2H, H-3, 5), 7.45 (t, J=7.7, 1H, H-10), 7.56 (d, J=7.7, 1H, H-8), 7.79 (t, J=7.7, 1H, H-9), 8.40 (d, J=7.7, 1H, H-11), 8.50 (t, J=5.3, 2H, H-2, 6), 13.94 (bs, 1H, NH); Anal. calcd. for C_16_H_9_FN_4_OS: C, 59.25; H, 2.80; N, 17.27; S, 9.89; Found: C, 59.22; H, 2.81; N, 17.28; S, 9.89.

#### 8-Methyl-3-phenyl-6-thioxo-6, 7-dihydro-2H-[1, 2, 4]triazino[2, 3-c]quinazolin-2-one (3.11)

Yield: 94%, mp >300°C; IR (cm^-1^): 3186, 3128, 3074, 3031, 2968, 1658, 1617, 1601, 1544, 1515, 1490, 1468, 1445, 1428, 1395, 1380, 1357, 1314, 1245, 1205, 1185, 1163, 1152, 1094, 1048, 1007, 997, 977, 933, 863, 838, 813, 800, 746, 689, 659, 619; ^1^H NMR δ= 3.07 (s, 1H, CH_3_), 7.57-7.40 (m, 4H, H-3', 4', 5', 10), 7.77 (d, 1H, J = 7.7, H-9), 8.33-8.17 (m, 3H, H-2', 6', 11,), 13.88 (s, 1H, NH); LC-MS, m/z = 321 [M+1], 323 [M+3] ; Anal. calcd. for C_17_H_12_N_4_OS: C, 63.73; H, 3.78; N, 17.49; S, 10.01; Found: C, 63.75; H, 3.79; N, 17.49; S, 10.02.

#### 9-Fluoro-3-phenyl-6-thioxo-6, 7-dihydro-2H-[1, 2, 4]triazino[2, 3-c]quinazolin-2-one (3.12)

Yield: 95%, mp >300°C; IR (cm^-1^): 3077, 3019, 2893, 2838, 1665, 1618, 1555, 1516, 1490, 1444, 1393, 1355, 1314, 1296, 1276, 1262, 1191, 1171, 1143, 1104, 1085, 1035, 1017, 1001, 977, 939, 860, 832, 814, 788, 776, 747, 709, 682, 659, 638, 618, 609; ^1^H NMR, δ: 7.25 (m, 2H, H-8, 10), 7.62-7.46 (m, 3H, H-3', 4', 5'), 8.38 (d, J=7.1, 2H, H-2', 6'), 8.50-8.43 (m, 1H, H-11), 13.94 (s, 1H, NH); LC-MS, m/z =325 [M+1]; Anal. calcd. for C_16_H_9_FN_4_OS: C, 59.25; H, 2.80; N, 17.27; S, 9.89; Found: C, 59.27; H, 2.80; N, 17.25; S, 9.86.

#### 9-Fluoro-3-(4-fluorophenyl)-6-thioxo-6, 7-dihydro-2H-[1, 2, 4]triazino[2, 3-c]quinazolin-2-one (3.13)

Yield: 92%, mp >300°C; IR (cm^-1^): 3096, 3057, 3016, 2894, 2809, 1651, 1625, 1598, 1558, 1502, 1471, 1411, 1391, 1354, 1335, 1295, 1272, 1234, 1184, 1166, 1143, 1103, 977, 942, 867, 841, 827, 802, 772, 752, 718, 703, 682, 672, 620; ^1^H NMR δ: 13.91 (bs, 1H, NH), 8.48 (t, J=5.3, 2H, H-2', 6'), 8.28 (t, J=7.0, 1H, H-11), 7.23 (m, 4H, H-8, 10, H-3',5'); LC-MS, m/z = 343 [M+1], 345 [M+3]; Anal. calcd. for C_16_H_8_F_2_N_4_OS: C, 56.14; H, 2.36; N, 16.37; S, 9.37; Found: C, 56.11; H, 2.36; N, 16.38; S, 9.38.

#### 9-Fluoro-3-(4-methoxyphenyl)-6-thioxo-6,7-dihydro-2H-[1, 2, 4]triazino[2, 3-c]quinazolin-2-one (3.14)

Yield: 91%, mp >300°C; IR (cm^-1^): 3016, 2878, 2837, 2788, 1659, 1601, 1569, 1551, 1538, 1505, 1488, 1457, 1436, 1419, 1398, 1355, 1304, 1267, 1189, 1178, 1168, 1107, 1081, 1029, 1006, 976, 938, 862, 838, 808, 798, 776, 765, 751, 721, 708, 676, 640, 618; ^1^H NMR (400 MHz) δ: 3.89 (s, 3H, 0CH_3_), 7.03 (d, J=8.0, 2H, H-3', 5'), 7.33-7.16 (m, 2H, H-8, 10), 8.53-8.33 (m, 3H, H-11, H-2', 6'), 13.91 (s, 1H, NH); LC-MS, m/z =355 [M+1], 357 [M+3]; Anal. calcd. for C_17_H_11_FN_4_O_2_S: C, 57.62; H, 3.13; N, 15.81; S, 9.05; Found: C, 57.63; H, 3.13; N, 15.80; S, 9.08.

#### 10-Chloro-3-phenyl-6-thioxo-6, 7-dihydro-2H-[1, 2, 4]triazino[2, 3-c]quinazolin-2-one (3.15)

Yield: 93%, mp >300°C; IR (cm^-1^): 3108, 3062, 2985, 2910, 1651, 1618, 1598, 1551, 1506, 1489, 1446, 1393, 1344, 1281, 1256, 1185, 1150, 1123, 1095, 1034, 1014, 1000, 963, 870, 827, 810, 792, 750, 689, 676, 651, 625; ^1^H NMR, δ: 7.62-7.40 (m, 4H, H-9, H-3', 4' 5'), 7.72 (d, J=7.3, 1H, H-8), 8.30 (s, 1H, H-11), 8.39 (d, J=6.9, 2H, H-2', 6'), 13.84 (s, 1H, NH); LC-MS, m/z =341 [M+1], 343 [M+3]; Anal. calcd. for C_16_H_9_CIN_4_OS: C, 56.39; H, 2.66; N, 16.44; S, 9.41; Found: C, 56.36; H, 2.66; N, 16.45; S, 9.44.

#### 10-Chloro-3-(4-methylphenyl)-6-thioxo-6, 7-dihydro-2H-[1, 2, 4]triazino[2, 3-c]quinazolin-2-one (3.16)

Yield: 93%, mp >300°C; IR (cm^-1^): 3416, 3308, 3063, 2988, 2917, 1652, 1616, 1599, 1549, 1516, 1503, 1456, 1405, 1386, 1344, 1331, 1310, 1255, 1185, 1164, 1152, 1139, 1123, 1094, 1015, 964, 872, 830, 820, 758, 731, 711, 697, 671, 650, 621, 607; ^1^H NMR, δ 2.46 (s, 3H, CH3), 7.32 (d, J = 7.8 Hz, 2H, H-3', 5'), 7.54 (d, J=8.7, 1H, H-8), 8.11 (d, J=7.5, 1H, H-9), 8.44-8.24 (m, 3H, H-11, H-2',6'), 13.95 (bs, 1H, NH); LC-MS, m/z = 355 [M+1], 357 [M+3], 359 [M+5]; Anal. calcd. for C_17_H_11_CIN_4_OS: C, 57.55; H, 3.12; N, 15.79; S, 9.04; Found: C, 57.58; H, 3.12; N, 15.77; S, 9.02.

#### 10-Chloro-3-(4-methoxyphenyl)-6-thioxo-6, 7-dihydro-2H-[1, 2, 4]triazino[2, 3-c]quinazolin-2-one (3.17)

Yield: 93%, mp >300°C; IR (cm^-1^): 3057, 2986, 2943, 2893, 2837, 1649, 1596, 1574, 1552, 1540, 1520, 1502, 1478, 1456, 1428, 1394, 1344, 1305, 1271, 1188, 1175, 1150, 1124, 1114, 1096, 1016, 1006, 965, 902, 872, 839, 821, 769, 759, 728, 699, 672, 629;^1^H NMR, δ: 3.89 (s, 3H, OCH3), 7.02 (d, J=8.6, 2H, H-3', 5'), 7.52 (d, J=8.7, 1H, H-8), 7.72 (d, J=8.6, 1H, H-9), 8.30 (s, 1H, H-11), 8.44 (d, J=8.6, 2H, H-2', 6'), 13.93 (s, 1H, NH); LC-MS, m/z =371 [M+1], 373 [M+3], 374 [M+4]; Anal. calcd. for C_17_H_11_CIN_4_S: C, 55.06; H, 2.99; N, 15.11; S, 8.65; Found: C, 55.09; H, 2.99; N, 15.10; S, 8.63.

#### 10-Chloro-3-(4-fluorophenyl)-6-thioxo-6, 7-dihydro-2H-[1, 2, 4]triazino[2, 3-c]quinazolin-2-one (3.18)

Yield: 90%, mp >300°C; IR (cm^-1^): 3478, 3062, 2986, 2885, 2838, 1658, 1599, 1556, 1516, 1503, 1478, 1453, 1427, 1412, 1393, 1344, 1323, 1284, 1262, 1239, 1189, 1159, 1122, 1101, 1012, 965, 874, 843, 832, 815, 776, 758, 733, 720, 689, 669, 656, 626, 610; ^1^H NMR, δ: 7.27 (t, J=8.7, 2H, H-3', 5'), 7.53 (d, J=8.8, 1H, H-8), 7.74 (d, J=8.8, 1H, H-9), 8.32 (s, 1H, H-11), 8.51 (dd, J^3^=8.6, J^4^=5.7, 2H, H-2',6'), 14.22 (bs, 1H, NH); LC-MS, m/z =359 [M+1], 361 [M+3]; Anal. calcd. for C_16_H_8_CIFN_4_OS: C, 53.56; H, 2.25; N, 15.62; S, 8.94; Found: C, 53.56; H, 2.25; N, 15.62; S, 8.94.

#### 8-Bromo-3-(4-fluorophenyl)-6-thioxo-6,7-dihydro-2H-[1, 2, 4]triazino[2, 3-c]quinazolin-2-one (3.19)

Yield: 92%, mp >300°C; IR (cm^-1^): 3473, 3327, 3065, 2974, 2787, 1597, 1587, 1550, 1530, 1514, 1473, 1428, 1413, 1379, 1334, 1320, 1287, 1259, 1230, 1161, 1101, 1089, 1055, 1024, 1015, 960, 924, 886, 843, 818, 787, 744,0 717, 699, 651, 626, 612; ^1^H NMR, δ: 6.64 (t, J=7.6, 1H, H-10), 7.23 (t, J=7.8, 2H, H-3', 5'), 7.62 (d, J=7.4, 1H, H-9), 7.76 (d, J=7.7, 1H, H-11), 8.30 (t, 2H, J=5.3, H-2',6'), 14.03 (bs, 1H, NH), LC-MS, m/z = 404 [M +1], 406 [M +3]; Anal. calcd. for C_16_H_8_BrN_4_OS: C, 47.66; H, 2.00; N, 13.89; S, 7.95; Found: C, 47.67; H, 2.01; N, 13.88; S, 7.94.

#### 9-Bromo-3-(4-fluorophenyl)-6-thioxo-6, 7-dihydro-2H-[1, 2, 4]triazino[2, 3-c]quinazolin-2-one (3.20)

Yield: 92%, mp >300°C; IR (cm^-1^): 3063, 2892, 1658, 1591, 1553, 1515, 1498, 1474, 1410, 1385, 1340, 1289, 1233, 1184, 1157, 1115, 1101, 1082, 1068, 1013, 944, 901, 872, 843, 823, 773, 751, 715, 699, 676, 664, 634, 622; ^1^H NMR, δ: 7.26 (t, J=8.5, 2H, H-3', 5'), 7.54 (d, J=8.5, 1H, H-10), 7.67 (s, 1H, H-8), 8.29 (d, J=8.5, 1H, H-11), 8.50 (dd, J1=7.5, J2=6.1, 2H, H-2', 6'), 13.79 (s, 1H, NH); LC-MS, m/z = 403 [M]; Anal. calcd. for C_16_H_8_BrFN_4_OS: C, 47.66; H, 2.00; N, 13.89; S, 7.95; Found: C, 47.68; H, 2.00; N, 13.90; S, 7.96.

#### 10-Bromo-3-phenyl-6-thioxo-6, 7-dihydro-2H-[1, 2, 4]triazino[2, 3-c]quinazolin-2-one (3.21)

Yield: 95%, mp >300°C; IR (cm^-1^): 1638, 1624, 1597, 1556, 1537, 1493, 1471, 1446, 1407, 1366, 1344, 1276, 1229, 1173, 1133, 1082, 1036, 1003, 990, 953, 859, 830, 817, 755, 699, 687, 669, 646; ^1^H NMR, δ: 7.63-7.37 (m, 4H, H-8, H-3', 4', 5'), 7.86 (d, J=8.7, 1H, H-9), 8.41 (d, J=6.9, 2H, H-2', 6'), 8.48 (s, 1H, H-11), 13.81 (s, 1H, NH), LC-MS, m/z = 387 [M+2]; Anal. calcd. for C_16_H_8_BrN_4_OS: C, 49.88; H, 2.35; N, 14.54; S, 8.32; Found: C, 49.85; H, 2.35; N, 14.56; S, 8.34.

#### 10-Bromo-3-(4-methylphenyl)-6-thioxo-6, 7-dihydro-2H-[1, 2, 4]triazino[2, 3-c]quinazolin-2-one (3.22)

Yield: 91%, mp >300°C; IR (cm^-1^): 3173, 3093, 3068, 2985, 2918, 1657, 1619, 1538, 1504, 1473, 1386, 1340, 1310, 1255, 1185, 1124, 1086, 1015, 955, 868, 832, 770, 755, 739, 710, 698, 684, 659; ^1^H NMR, δ: 2.46 (s, 3H, CH3), 7.31 (d, J=7.7, 1H, H-3', 5'), 7.47 (d, J=8.7, 1H, H-8), 7.86 (d, J=8.7, 1H, H-9), 8.31 (d, J=7.6, 2H, H-2', 6'), 8.46 (s, 1H, H-11), 13.91 (bs, 1H, NH); LC-MS, m/z = 399 [M], 403 [M+4]; Anal. calcd. for C_17_H_11_BrN_4_OS: C, 51.14; H, 2.78; N, 14.03; S, 8.03; Found: C, 51.17; H, 2.78; N, 14.04; S, 8.05.

#### 10-Bromo-3-(4-fluorophenyl)-6-thioxo-6, 7-dihydro-2H-[1, 2, 4]triazino[2, 3-c]quinazolin-2-one (3.23)

Yield: 95%, mp >300°C; IR (cm^-1^): 3478, 3173, 3065, 2985, 2888, 1650, 1598, 1555, 1516, 1501, 1475, 1453, 1411, 1384, 1340, 1323, 1299, 1281, 1257, 1236, 1186, 1158, 1124, 1101, 1085, 1015, 958, 882, 869, 841, 814, 776, 757, 738, 718, 695, 659, 625, 608; ^1^H NMR, δ: 7.27 (t, J=8.7, 2H, H-3', 5'), 7.48 (d, J=8.6, 1H, H-8), 7.87 (dd, J=8.7, J2=1.6, 1H, H-9), 8.47 (s, 1H, H-11), 8.55 (dd, J1=8.0, J2=6.1,2H, H-2', 6'), 13.87 (s, 1H, NH); LC-MS, m/z =403 [M]; Anal. calcd. for C_16_H_8_BrFN_4_OS: C, 47.66; H, 2.00; N, 13.89; S, 7.95; Found: C, 47.69; H, 2.00; N, 13.89; S, 7.97.

#### 10-Bromo-3-(4-methoxyphenyl)-6-thioxo-6, 7-dihydro-2H-[1, 2, 4]triazino[2, 3-c]quinazolin-2-one (3.24)

Yield: 94%, mp >300°C; IR (cm^-1^): 3090, 3056, 2986, 2941, 2911, 1647, 1626, 1606, 1593, 1573, 1552, 1541, 1520, 1499, 1474, 1455, 1440, 1425, 1391, 1353, 1342, 1304, 1270, 1258, 1236, 1187, 1175, 1151, 1114, 1087, 1016, 959, 903, 870, 839, 820, 768, 759, 736, 698, 685, 659, 628; ^1^H NMR, δ: 3.89 (s, 3H, OCH3); 7.03 (d, J=8.7, H-3', 5'), 7.46 (d, J=8.8, 1H, H-8), 7.85 (d, J=8.6, 1H, H-9), 8.52-8.38 (m, 3H, H-11, H-2', 6'), 13.86 (bs, 1H, NHz); LC-MS, m/z = 415 [M]; Anal. calcd. for C_17_H_11_BrN_4_O_2_S: C, 49.17; H, 2.67; N, 13.49; S, 7.72; Found: C, 49.19; H, 2.67; N, 13.48; S, 7.74.

#### 10-lodo-3-(4-fluorophenyl)-6-thioxo-6, 7-dihydro-2H-[1, 2, 4]triazino[2, 3-c]quinazolin-2-one (3.25)

Yield: 93,%, mp >300°C; IR (cm^-1^): 3496, 3358, 2886, 1678, 1628, 1609, 1590, 1554, 1538, 1521, 1505, 1488, 1472, 1444, 1403, 1375, 1337, 1328, 1297, 1276, 1241, 1215, 1172, 1158, 1126, 1100, 1075, 1009, 992, 951, 844, 831, 824, 815, 772, 759, 747, 718, 687, 622, 605; ^1^H NMR, δ: 6.69 (d, J=8.7, 1H, H-8); 7.18 (t, J=8.6, 2H, H-3', 5'), 7.41 (d, J=8.7, 1H, H-9), 8.07 (s, 1H, H-11), 8.31 (t, J=8.7, 2H, H-2', 6'), LC-MS, m/z =450 [M], 452 [M+2]; Anal. calcd. for C_16_H_8_FIN_4_OS: C, 42.68; H, 1.79; N, 12.44; S, 7.12; Found: C, 42.67; H, 1.79; N, 12.46; S, 7.14.

#### 10-lodo-3-(4-methoxyphenyl)-6-thioxo-6, 7-dihydro-2H-[1, 2, 4]triazino[2, 3-c]quinazolin-2-one (3.26)

Yield: 93%, mp >300°C; IR (cm^-1^): 3078, 3019, 2974, 2896, 2836, 1622, 1606, 1590, 1571, 1552, 1533, 1511, 1472, 1445, 1435, 1421, 1405, 1368, 1338, 1306, 1284, 1270, 1243, 1173, 1139, 1124, 1112, 1078, 1030, 1010, 992, 951, 880, 861, 837, 830, 806, 770, 723, 702, 652, 628; ^1^H NMR: δ: 3.94 (s, 3H, OCH_3_); 7.14 (d, J=8.2, H-3', 5'), 7.51 (d, J=8.5, 1H, H-8), 7.91 (d, J=8.6, 1H, H-9), 8.59-8.42 (m, 3H, H-11, H-2', 6'), 13.92 (s, 1H,NH); LC-MS, m/z =463 [M+1], 465 [M+3]; Anal. calcd. for C_17_H_11_IN_4_O_2_S: C, 44.17; H, 2.40; N, 12.12;S, 6.94; Found: C, 44.19; H, 2.40; N, 12.11; S, 6.92.

### Pharmacology

#### Antimicrobial Test

The sensitivity of the microorganisms to the synthesized compounds was evaluated according the described methods [8, 9]. The assay was conducted on Mueller-Hinton medium by two-fold serial dilution of the compound in 1 mL, after that 0.1 mL of microbial seeding (10^6^ cells/mL) was added. The minimal inhibitory concentration of the compound was determined by the absence of visual growth in the test tube with a minimal concentration of the substance, then the minimal bactericide/fungicide concentration was determined by the absence of growth on agar after inoculation of the microorganism from the transparent test tubes. Dimethylsulfoxide was used as a solvent, with an initial solution concentration of 1 mg/mL. Preliminary screening was performed on *Staphylococcus aureus* ATCC 25923, *Escherichia coli* ATCC 25922, *Pseudomonas aeruginosa* ATCC 27853, and *Candida albicans* ATCC 885-653 standard test cultures. All test strains were received from the bacteriological laboratory Zaporizhzhya Regional Laboratory Center of State Sanitary and Epidemiological Service of Ukraine. Nitrofual and Trimetoprim were used as the reference compound with proven antibacterial/antifungal activity. Additional quality control of the culture medium and solvents was conducted by commonly used methods [17].

## Conclusion

In the present paper, 43 new 8-R^1^-9-R^2^-10-R^3^-3-R-6-thio-6, 7-dihydro-2H-[1, 2, 4]triazino-[2, 3-*c*]quinazoline-2-ones and their potassium salts were described. The synthesized compounds were tested for antibacterial and antifungal activity. As a result of the microbiological assay, the compounds with high inhibitory action against *Staphylococcus aureus* ATCC 25923 (MIC 6.25–100 μg/mL, MBC 12.5–200 μg/mL) were found.
